# Cross-sectional assessment of factors associated with functional capacity in patients with low back pain attending family medicine outpatient clinics

**DOI:** 10.1590/1806-9282.20260621

**Published:** 2026-10-30

**Authors:** İsmail Arslan, Şeyma Ceylan, Duygu Yengil Taci

**Affiliations:** 1 Ankara Training and Research Hospital, Family Medicine Clinics – Ankara, Turkey.

**Keywords:** Low back pain, Functional impairment, Disability, Daily activities

## Abstract

**OBJECTIVE:**

Low back pain is a common health problem that may lead to functional limitations and disability, restricting daily activities, reducing work productivity, and adversely affecting social participation and quality of life. This study aimed to evaluate the factors associated with functional capacity in patients with low back pain attending family medicine outpatient clinics.

**METHODS:**

This single-centre, cross-sectional study included 250 patients aged 18–65 years with chronic low back pain. Data were collected through face-to-face interviews using a structured questionnaire covering sociodemographic characteristics, chronic diseases, medication use, and low back pain characteristics. Pain intensity was assessed using the Visual Analog Scale, and functional capacity was assessed using the Back Pain Functional Scale.

**RESULTS:**

In the univariable analyses, lower Back Pain Functional Scale scores were observed in women, participants aged ≥55 years, individuals with obesity, housewives, those with lower educational attainment and income, and those with chronic diseases (all p<0.05). Back Pain Functional Scale scores were negatively correlated with pre-treatment Visual Analog Scale scores (Spearman's ρ=−0.419, p<0.001). In the multivariable analysis, male sex was independently associated with higher Back Pain Functional Scale scores, whereas higher body mass index, greater pain intensity, and reporting heavy lifting as the aggravating factor were independently associated with lower Back Pain Functional Scale scores (p<0.05).

**CONCLUSION:**

This study identified several factors associated with functional capacity in patients with low back pain. Higher body mass index, greater pain intensity, and heavy lifting as an aggravating factor were independently associated with poorer functional capacity. These findings may help clinicians identify patients at increased risk of functional impairment and support comprehensive assessment and management of low back pain.

## INTRODUCTION

Low back pain (LBP) is one of the most common reasons for seeking healthcare, particularly in family medicine outpatient clinics. It is a multifactorial condition with diverse underlying causes, and its impact on daily functioning varies considerably among individuals. Psychological, social, and biological factors, together with comorbidities, contribute to pain and functional impairment^1^. As one of the leading causes of disability worldwide, LBP is the second most common reason for consultations with family physicians in primary care^2^. Approximately 80% of individuals experience LBP at least once during their lifetime, whether resulting from traumatic or non-traumatic causes. In 10–20% of adults, LBP becomes chronic, leading to persistent functional limitations and difficulties returning to work^3^. Individuals with LBP commonly report difficulties performing routine tasks and activities of daily living^4^. Functional limitations generally increase with greater pain intensity and older age, with the greatest restrictions observed in activities involving heavy lifting, walking, and prolonged standing. In addition, LBP frequently disrupts sleep, reduces energy levels, and adversely affects social participation^5^. Beyond its physical effects, LBP is also associated with emotional distress and reduced quality of life. Effective management of LBP requires a multidisciplinary approach that combines pharmacological and non-pharmacological interventions. Pharmacological treatment may include acetaminophen, non-steroidal anti-inflammatory drugs, muscle relaxants, opioids, and antidepressants, while non-pharmacological approaches include physical therapy and cognitive-behavioral therapy^6^. Additional non-pharmacological interventions include patient education, exercise, massage, acupuncture, lumbar schools, and kinesiology taping^6^.

Given the substantial impact of LBP on health and daily functioning, identifying the factors associated with functional capacity is important for optimizing patient management in primary care. Therefore, this cross-sectional study aimed to evaluate the factors associated with functional capacity among patients with LBP attending family medicine outpatient clinics, including sociodemographic characteristics and pain-related clinical features, such as pain onset, intensity, duration, frequency, triggering factors, and associated symptoms. Functional capacity was assessed using the Back Pain Functional Scale (BPFS), developed by Stratford et al.^7^, which was validated and demonstrated to be reliable in the Turkish population in 2017^8^.

## METHODS

This single-center, cross-sectional study included 250 patients aged 18–65 years who attended the Family Medicine outpatient clinic at Ankara Training and Research Hospital with complaints of LBP. Eligible participants had chronic LBP (pain lasting ≥3 months) due to non-infectious, non-inflammatory, and non-neoplastic causes. Patients with LBP secondary to infectious, inflammatory, visceral, neoplastic, traumatic, fracture-related, or metabolic conditions were excluded, as were individuals with communication difficulties, those who declined to participate, or those who returned incomplete questionnaires. Ethical approval was obtained from the Ethics Committee of Ankara Training and Research Hospital (Date: 06.09.2023; Decision No: 1376).

An a priori sample size calculation was performed using the formula for a finite population (n=1,500). Assuming a 95% confidence level and a 5% margin of error, the minimum required sample size was calculated as 230 participants. To compensate for potential incomplete data and missing responses, the target sample size was increased by approximately 10%, resulting in a final sample of 250 participants.

The study questionnaire was developed by the researchers based on a review of the relevant literature and included questions on sociodemographic characteristics, chronic diseases, medication use, the onset and duration of LBP, and factors that aggravated or alleviated pain. Pain intensity was assessed using a 10-cm Visual Analog Scale (VAS), ranging from 0 ("no pain") to 10 ("the worst pain imaginable"), with higher scores indicating greater pain intensity^9^. Functional capacity was assessed using the BPFS, developed by Stratford et al. and validated for use in the Turkish population in 2017^8^. The BPFS consists of 12 items with a total score ranging from 0 to 60, where higher scores indicate better functional capacity and lower levels of disability.

Data were analyzed using IBM SPSS Statistics version 25.0 (IBM Corp., Armonk, NY, USA). Continuous variables are presented as mean±standard deviation (SD), and categorical variables as frequencies and percentages. The normality of continuous variables was assessed using the Shapiro-Wilk test. Depending on data distribution, comparisons were performed using the independent-samples t-test, one-way analysis of variance, Mann-Whitney U test, or Kruskal-Wallis test. Spearman's rank correlation coefficient was used to assess the association between BPFS and VAS scores. A multivariable linear regression model was constructed to identify factors independently associated with BPFS scores. A two-sided p<0.05 was considered statistically significant.

## RESULTS

Out of the 250 participants, 95 (38.0%) were male, and 155 (62.0%) were female. The mean BPFS score for the study population was 36.12±13.27. In the univariable analyses, lower BPFS scores were significantly associated with female sex, age ≥55 years, obesity, being a housewife, primary school education, lower income, and the presence of chronic disease (all p<0.05). BPFS scores according to the sociodemographic characteristics of the participants are presented in Table 1.

**Table 1:** The Back Pain Functional Scale scores of the study population categorized by sociodemographic characteristics.

Sociodemographic characteristics		n (%)	BPFS score Mean±SD	p-value
Age (years)				
	<40	92 (36.8)	42.55±11.83	<0.001*
	40–54	79 (31.6)	35.39±11.89	
	≥55	79 (31.6)	32.09±14.78	
Gender				
	Female	155 (62.0)	34.10±13.68	<0.001**
	Male	95 (38.0)	41.69±12.00	
BMI categories				
	Normal	91 (36.4)	38.79±14.04	<0.001*
	Overweight	85 (34.0)	40.32±11.88	
	Obese	74 (29.6)	30.93±12.93	
Level of education				
	Primary or below	69 (27.6)	29.75±13.41	<0.001**
	High school	50 (20.0)	36.20±11.76	
	University	131 (52.4)	41.09±12.68	
Occupation				
	White collar	111 (44.4)	41.21±13.00	<0.001*
	Blue collar	66 (26.4)	37.89±11.92	
	Housewife-no work	73 (29.2)	29.74±12.95	
Employment status				
	Full time	150 (60.0)	39.84±12.23	<0.001*
	Retired	25 (10.0)	35.72±17.63	
	Housewife	75 (30.0)	31.69±13.07	
Income				
	Low	70 (28.0)	33.27±13.41	<0.001*
	Medium	121 (48.4)	35.71±13.62	
	High	59 (23.6)	44.00±10.99	
Smoking status				
	Current smoker	74 (29.6)	37.08±12.62	0.815*
	Former smoker	35 (14.0)	37.94±15.58	
	Never smoker	141 (56.4)	36.70±13.58	
Presence of chronic disease				
	Yes	115 (46.0)	32.94±14.54	<0.001**
	No	135 (54.0)	40.30±11.69	
Continuous medication use				
	Yes	105 (42.0)	33.42±14.42	0.001**
	No	145 (58.0)	39.57±12.32	

SD: standard deviation; BPFS: Back Pain Functional Scale; BMI: body mass index.

* Kruskal-Wallis test.

** Mann-Whitney U test p<0.05 is statistically significant.

The mean VAS score was 6.67±2.30. A significant moderate negative correlation was observed between VAS and BPFS scores (Spearman's ρ=-0.419, p<0.001).

The most commonly reported triggering factors for LBP were prolonged maintenance of the same position (54.8%) and heavy lifting (32.4%). Participants reporting falls or heavy lifting as the triggering factor had significantly lower BPFS scores than those reporting other triggering factors (p=0.020).

Prolonged standing (43.6%) was the most frequently reported aggravating factor, whereas rest (66.4%) was the most frequently reported alleviating factor. BPFS scores according to LBP characteristics are presented in Table 2.

**Table 2:** The Back Pain Functional Scale scores of the study population according to the characteristics of low back pain.

Characteristics of LBP		n (%)	BPFS score Mean±SD	p-value
Factors that trigger the onset of LBP				
	Heavy lifting	81 (32.4)	33.52±15.14	0.020*
	Staying in the same position for a prolonged time	137 (54.8)	39.41±12.45	
	Falls	14 (5.6)	31.07±14.69	
	Pregnancy	18 (7.2)	38.72±8.48	
Factors that increase LBP (aggravating factors for LBP)				
	Heavy lifting	60 (24.0)	31.65±13.48	0.001*
	Prolonged standing	109 (43.6)	39.10±13.05	
	Prolonged sitting	38 (15.2)	41.92± 11.98	
	Other	43 (17.2)	34.70± 13.79	
Factors that reduce LBP (alleviating factors)				
	Rest	166 (66.4)	36.23±13.77	0.163*
	Exercise	46 (18.4)	40.20±12.35	
	Lying on your back	38 (15.2)	36.39± 13.78	

SD: standard deviation; BPFS: Back Pain Functional Scale; LBP: Low back pain.

* Kruskal Wallis test. p<0.05 is statistically significant.

BPFS scores were initially evaluated using univariable analyses (Table 3). The variables examined included age, sex, body mass index (BMI), educational level, presence of chronic disease, pre-treatment pain intensity (VAS score), factors triggering the onset of LBP (heavy lifting, prolonged maintenance of the same position, falls, and pregnancy), aggravating factors (heavy lifting, prolonged standing, prolonged sitting, and other), and alleviating factors (rest, exercise, and lying in the supine position).

**Table 3:** Predictors of Back Pain Functional Scale score: Univariable regression analysis.

Predictor		Univariable B (95%CI)	p
Age (years)		-0.36 (-0.48 to -0.24)	<0.001
Male sex (vs. female)		7.60 (4.24–10.95)	<0.001
BMI (kg/m^2^)		-0.75 (-1.07 to -0.43)	<0.001
Educational level			
	High school (vs. primary school or below)	6.45 (1.80–11.10)	0.007
	University (vs. primary school or below)	11.35 (7.61–15.09)	<0.001
	No chronic disease (vs. yes)	7.20 (3.93–10.47)	<0.001
	VAS score	-2.53 (-3.19 to -1.86)	<0.001
Triggering factors			
	Prolonged static position (vs. heavy lifting)	5.89 (2.22–9.56)	0.002
	Fall (vs. heavy lifting)	-2.45 (-10.03 to 5.13)	0.525
	Pregnancy (vs. heavy lifting)	5.20 (-1.62 to 12.02)	0.134
Aggravating factors			
	Heavy lifting as aggravating factor (vs. prolonged standing)	-7.45 (-11.60 to -3.29)	<0.001
	Prolonged sitting (vs. prolonged standing)	2.82 (-2.05 to 7.69)	0.255
	Other aggravating factor (vs. prolonged standing)	-4.40 (-9.06 to 0.26)	0.064
Alleviating factors			
	Exercise (vs. rest)	3.97 (-0.47 to 8.41)	0.080
	Supine position (vs. rest)	0.17 (-4.63 to 4.96)	0.946

BMI: body mass index; CI: confidence interval; VAS: Visual Analog Scale.

A multivariable linear regression model was subsequently developed to identify factors independently associated with BPFS scores (Table 4). Variables considered clinically relevant, together with those showing an association with BPFS scores in the univariable analyses, were entered into the model. Categorical independent variables were coded as dummy variables, with clinically relevant reference categories used for comparison.

**Table 4:** Predictors of Back Pain Functional Scale score in multivariate regression analysis.

Variables	Standardized beta coefficient	Odds ratio (95%CI)	p
Male sex (vs. female)	0.196	5.46 (2.19 to 8.73)	0.001
BMI (kg/m^2^)	-0.138	-0.37 (-0.69 to -0.04)	0.028
VAS score	-0.259	-1.52 (-2.24 to -0.81)	<0.001
Heavy lifting	-0.130	-4.12 (-8.08 to -0.15)	0.042

BMI: body mass index; CI: confidence interval; VAS: Visual Analog Scale.

The overall model was statistically significant [F(15,234)=7.67, p<0.001], explaining 33.0% of the variance in BPFS scores (R^2^=0.330; adjusted R^2^=0.287).

After adjustment for potential confounders, male sex was independently associated with higher BPFS scores (B=5.46, 95%CI 2.19–8.73; standardized β=0.196; p=0.001). Conversely, higher BMI (B=-0.37, 95%CI -0.69 to -0.04; standardized β=-0.138; p=0.028) and greater pre-treatment pain intensity (VAS score) (B=-1.52, 95%CI -2.24 to -0.81; standardized β=-0.259; p<0.001) were independently associated with lower BPFS scores. Furthermore, reporting heavy lifting as the aggravating factor was independently associated with lower BPFS scores compared with prolonged standing (B=-4.12, 95%CI -8.08 to -0.15; standardized β=-0.130; p=0.042). Age, educational level, chronic disease, triggering factors, and alleviating factors were not independently associated with BPFS scores (all p>0.05).

Assessment of multicollinearity demonstrated no evidence of problematic collinearity among the independent variables. Variance inflation factor values ranged from 1.08 to 2.31, indicating that multicollinearity was not a concern in the final regression model.

## DISCUSSION

This cross-sectional study evaluated factors associated with functional capacity among patients with LBP attending family medicine outpatient clinics. In the multivariable analysis, male sex was independently associated with higher BPFS scores, whereas higher BMI, greater pre-treatment pain intensity, and reporting heavy lifting as the aggravating factor were independently associated with lower BPFS scores. In the univariable analyses, lower BPFS scores were also observed among older participants, housewives, individuals with chronic disease, and those with lower educational attainment and income. These findings highlight the multifactorial nature of functional impairment in patients with LBP and emphasize that both clinical and sociodemographic characteristics should be considered when evaluating patients with LBP^10,11^.

The onset of LBP caused by falls and heavy lifting was associated with worse functional outcomes in the univariable analyses, supporting previous research suggesting that mechanical injuries to the spine may result in greater functional impairment^12^. Additionally, a previous study reported that rest provided the most immediate relief for LBP, whereas physical activity and exercise were beneficial for long-term management^13^. Understanding the factors associated with reduced functional capacity may help clinicians identify patients at greater risk of functional impairment and tailor management strategies accordingly.

Our study demonstrated a significant sex difference in functional capacity, with women exhibiting lower BPFS scores than men. Consistent with previous studies, reduced lumbar function has been reported more frequently among women^12,13^. Although the underlying mechanisms were not directly assessed in the present study, one possible explanation is that women generally have lower muscle mass and reduced spinal support than men, as suggested by previous research. In addition, postpartum changes, including weakened abdominal and paraspinal muscles, may further increase the risk of functional limitation^14^. These findings highlight the potential importance of considering sex-specific factors when planning rehabilitation strategies for patients with LBP.

Lower BPFS scores were also observed among older participants in the univariable analyses. Previous studies suggest that age-related changes in muscles, bones, and joints contribute to declining physical function and increased disability^5,15^. For example, a 2022 study reported that individuals aged 35 years and older were more likely to experience limitations in daily activities than younger adults^10^. Increased pain severity and reduced physical performance with advancing age may further contribute to functional limitations^16,17^. However, age was not independently associated with BPFS scores after adjustment for other variables, suggesting that its apparent effect may be partly explained by other clinical or demographic characteristics.

Housewives had lower BPFS scores than blue-collar and white-collar workers in the univariable analyses. Previous studies have suggested that physically demanding household activities, prolonged awkward postures, and repetitive tasks may contribute to LBP and functional limitations^18^. Similarly, both housewives and blue-collar workers may be at increased risk of functional impairment because of heavy lifting, demanding work conditions, and suboptimal ergonomic practices. However, these occupational exposures were not directly assessed in our study.

Lower educational attainment and lower income were associated with poorer functional capacity in the univariable analyses, consistent with previous studies^19-24^. This relationship may reflect differences in occupational demands, health literacy, access to healthcare, and working conditions among individuals with lower socioeconomic status. However, educational level and income were not independently associated with BPFS scores in the multivariable analysis, suggesting that these associations may be influenced by other demographic and clinical characteristics.

Consistent with previous research, higher BMI was independently associated with lower BPFS scores in the multivariable analysis^25-27^. Excess body weight may increase mechanical loading of the spine and contribute to metabolic alterations that adversely affect musculoskeletal tissues^28^. Given the increasing prevalence of obesity worldwide, weight management should be considered an important component of comprehensive LBP management.

Participants with chronic diseases had lower BPFS scores in the univariable analyses. Previous studies have shown that chronic diseases frequently coexist with LBP and may contribute to reduced physical function and difficulties in daily activities^29,30^. This relationship may be explained by deconditioning syndrome, characterized by physiological changes associated with reduced physical activity, which can exacerbate pain and functional limitations in individuals with comorbidities. However, chronic disease was not independently associated with BPFS scores after adjustment for other variables.

Consistent with the well-established role of pain intensity as a determinant of functional disability in patients with chronic LBP, we observed a significant negative correlation between pre-treatment VAS scores and BPFS scores. Furthermore, higher pain intensity remained independently associated with lower BPFS scores in the multivariable analysis. This finding is consistent with that of Kose and Hatipoglu, who also reported that greater pain intensity was associated with more severe functional disability in patients with lumbar disc herniation^11^. Although the underlying mechanisms were not directly assessed in our study, previous research suggests that pain may reduce functional capacity through altered movement patterns, increased spinal loading, and pain-related fear of movement (kinesiophobia). From a clinical perspective, routine assessment of pain intensity may help identify patients at greater risk of functional decline and facilitate timely rehabilitative interventions.

Previous studies have identified prolonged sitting, prolonged standing, static postures, forward bending, and heavy lifting as important occupational risk factors for LBP^18,31^. In our study, the most frequently reported triggering factors were prolonged maintenance of the same position and heavy lifting. This discrepancy may be explained by the broader categorization used in our study, in which prolonged standing, sitting, and forward bending were grouped under prolonged maintenance of the same position.

Participants reporting falls or heavy lifting as the triggering factor had lower BPFS scores in the univariable analyses. In addition, reporting heavy lifting as the aggravating factor remained independently associated with lower BPFS scores in the multivariable analysis. These findings are consistent with previous studies demonstrating that repetitive mechanical loading of the spine contributes to functional impairment and disability^32,33^. They further highlight the importance of ergonomic interventions and preventive strategies aimed at reducing excessive spinal loading.

Consistent with previous studies, longer pain duration was associated with poorer quality of life and reduced physical function^34-36^. Chronic pain may adversely affect physical health, occupational performance, and social participation, thereby contributing to progressive functional decline.

Although participants who reported exercise as an alleviating factor had numerically higher BPFS scores than those reporting other alleviating factors, this difference did not reach statistical significance. Nevertheless, previous studies have shown that exercise may reduce pain and disability in individuals with chronic LBP^37-40^, possibly by improving posture, reducing muscle spasm, strengthening core musculature, and enhancing aerobic capacity. Therefore, while our findings do not demonstrate a significant association between exercise and functional capacity, they remain consistent with the broader body of evidence supporting exercise as an important component of LBP management.

LBP continues to represent a major public health problem worldwide because of its substantial impact on productivity and daily functioning^20,21^. The strength of our study lies in identifying factors associated with functional capacity among patients with LBP in a primary care setting. However, several limitations should be acknowledged. First, this was a single-center, cross-sectional study. Therefore, causal relationships between the evaluated factors and functional capacity cannot be established, and the findings should be interpreted as associations rather than cause-and-effect relationships. Furthermore, the single-center nature of the study may limit the generalisability of the findings to other populations and healthcare settings. Second, participants were recruited from a single family medicine outpatient clinic, which may have introduced selection bias, as individuals seeking medical care may differ from individuals with LBP in the general population who do not seek treatment. Third, information on pain characteristics and aggravating or alleviating factors was obtained through self-reported questionnaires, making the data susceptible to recall and reporting bias, particularly for symptoms experienced over extended periods. Although multivariable analyses were performed, residual confounding due to unmeasured or unknown factors cannot be excluded. Finally, the inclusion of patients at different stages and durations of LBP may have contributed to heterogeneity in functional capacity and influenced the observed associations.

## CONCLUSION

This cross-sectional study identified several factors associated with functional capacity among patients with LBP attending family medicine outpatient clinics. In the multivariable analysis, male sex was independently associated with higher functional capacity, whereas higher BMI, greater pain intensity, and reporting heavy lifting as the aggravating factor were independently associated with lower functional capacity. These findings highlight the multifactorial nature of functional impairment in patients with LBP and underscore the importance of comprehensive clinical assessment, including both sociodemographic and pain-related characteristics. Future prospective longitudinal studies are warranted to clarify causal relationships and evaluate interventions aimed at improving functional outcomes in patients with LBP.

## Data Availability

The datasets generated and/or analyzed during the current study are available from the corresponding author upon reasonable request.
